# Disaster recovery and structural inequalities: A case study of community assertion for justice

**DOI:** 10.1016/j.ijdrr.2021.102555

**Published:** 2021-12

**Authors:** Jacquleen Joseph, S. Mohammed Irshad, Allan Mathew Alex

**Affiliations:** Jamsetji Tata School of Disaster Studies, Tata Institute of Social Sciences, Mumbai, India

**Keywords:** Disaster recovery, Landlessness, Marginalization, Structural inequality, Injustice

## Abstract

Formal interventions are rationalized to be irreplaceable, especially with marginalized communities that are presumed to lack capacity. It is event centric and differ considerably from the community's experience of disaster risk and recovery within the everyday context. Thus, community engagement with multiple formal institutions that often fail to address recovery needs of the most marginalized, is inevitable. These contradictions lead to varied forms of community assertion towards addressing structural inequalities and injustices. In this paper we explore these contradictions by drawing from the work of scholars who recognize the limits of procedural justice and push for distributive justice, especially by focusing on grassroots processes using the lens of the politics of neo-liberalism and ontology of possibilities. Using a multi-sited instrumental case study approach the paper explores community's lived experiences, factors contributing to the persistence of structural inequality and injustice, and the alternate conception of justice and their assertions, in the disaster recovery context. The two case studies - Vistapit Mukti Vahini and Thayillam, inform an alternate theoretical conception of disaster recovery embedded in structural inequalities and injustices through the following three perspectives: Firstly how disaster risk and recovery emerge from historical and everyday lived reality of marginalized communities, their social relations and resulting material conditions; Secondly how challenging everyday social relations, processes and injustices is central to the community's alternate conception and assertion for disaster recovery; and finally how community assertion and recovery relies on the mobilization of vulnerability, which could mean being exposed and agentic at the same time.

## 1 Introduction

Over the past decade the research focus on disaster recovery has considerably increased, however, it continues to be relatively less explored in comparison to other phases of a disaster. The focus within disaster recovery research had shifted from the individual subject to that of the larger macro systems like economic and regional recovery over the years and, the preoccupation with rebuilding and reconstruction by formal actors continue to dominate the recovery literature [1]. Further with the onslaught of the resilience discourse there is a preoccupation with bouncing back or building back better and the potential for enhancing community resilience in the recovery context. Though there is a clear recognition of the fact that disaster recovery is a social process and not merely a reestablishment of physical and built environment, the focus on the social aspect has been limited to post-disaster processes and not much attention is given to the pre-disaster conditions of inequality and vulnerability that differentially expose communities to disasters and impact their capacities to recover. Although the critical literature on disaster vulnerability and resilience squarely question issues of structural inequality and violence, the discourse is at a nascent phase in disaster recovery research and practice. The critical literature on recovery in the context of economic depression [2], problematize the therapeutic conceptualization of recovery and argues for a forensic or a legal conceptualization. Drawing from the biological analogy of health the therapeutic conceptualization of recovery signifies a transition from an unsound/pathological condition to a sound/normal state of affairs. The assumption of a normal state of affair is problematized and the application of the biological analogy on to a social process raised several unanswered questions, such as: What period of the past was the community/society in good health? How to discover the natural causes of this pathological state? How to differentiate the cause from the symptom? and What constitutes an effective cure? among others. In the forensic conceptualization of recovery, the idea of right is fundamental and the focus in not on what a society is and what it was, but rather on what it should be. Thus, the conceptualization of recovery in the sense of the rehabilitation of the old or revival of the good old days is rejected in support of reform. Reforms envisage a “new” social order and urges to pursue untried courses of action that change the underlying conditions that thwart full attainment of normal well-being, in the face of unprecedented problems and issues [2]. Though the idea of rights and its realization in itself if debatable [3,4], the complete absence of the rights discourse in the disaster recovery conceptualization is a significant gap. The review of literature on disaster recovery, especially the definitions do not reflect the idea of right. Table 1 given below captures some of these recovery conceptions that go beyond repair, restoration, rebuilding/reconstruction and rehabilitation, among others. The closest that they get to be is the idea of build back better, which often does not engage with issues of structural inequality and justice.

**Table 1:** Conceptualizations of disaster recovery.

Sl. No.	Conceptualization of disaster recovery	Author, Year
1	Disaster recovery is expected to anticipate long-term losses and also concerned about recreation of pre-disaster trend.	[5]
2	Recovery should not define as a project of bouncing back, such generalization often neglects all other external factors influencing disaster recovery.	[6]
3	Disaster recovery is considered as a dynamic process in which it creates, maintain and change the meaning of the life of the survivors and it reaches to conclusion when the survivors move to redevelop and attain self-reliance.	[7]
4	Recovery process focuses on community as an agent of change. The traditional practices of community considered as social institutions are assigned the responsibility to initiate the project of disaster recovery	[8]
5	Disaster recovery should be people focused and it should engage the people in decision making to empower the community.	[51]
6	Disaster recovery is not merely a reestablishment of physical and built environment, rather it is about a social process which enable the community to involve in decision making process.	[9]

A review of literature on disaster recovery and structural inequalities too bring out the gap in literature with regard to the rights perspective. Research literature that directly address issues of structural inequality and injustice in disaster recovery is minuscule and limited to either description of issues of structural inequality [10], or developing theoretical frameworks that could be used to operationalize justice in disaster practice [11,12]. The framework for just recovery, principles of justice and sustainable or holistic recovery are among these [[11], [12], [50]]). Disaster recovery practice on the other hand co-opt issues of justice and inequality in the policy and practice arena by primarily developing procedural guidelines and frameworks that focus on accountability, transparency and participation, among others [13,14]. A good number of disaster research primarily highlight the significance of procedural aspects for operationalizing justice like for e.g. the significance of community participation [15], participatory processes [16], community decision making [17] and attributes of a community like social capital, networks, empowerment and assets [18] that are key to rights and justice claims. Though the available literature frames ‘community as agents of change’, most literature focus on the processes to be followed by formal actors or the attributes of the community that facilitate involvement in recovery processes. Within the broader literature on social justice there has been a push towards de-emphasizing or shifting the gaze from the attributes of people and systems to that of power relations and social processes that contribute to these attributes [19,20]. Moreover, there have been very few scholars who have focused on community's initiatives and movements to address issues of inequality and injustice in the post-disaster recovery context [[21], [22], [23]]. Thus, the mainstream disaster recovery research and practice is preoccupied with procedural aspects of operationalizing justice with a sole focus on formal humanitarian and bureaucratic actors.

The skewed focus on formal sector actors in disaster recovery especially in the South Asian context is attributed to the community's lack of capacity or levels of deprivation that necessitate external institutional interventions [24]. Thus, in the Indian context too disaster recovery is still a bureaucratic and technocratic project and bureaucracy among other formal actors defines and decides what recovery would entail. Often this conceptualization is limited to rebuilding resilient homes and rarely engages with issues of pre-disaster structural inequality and violence. The hegemony of formal actors and the technocratic approach appropriates issues of justice by creating one-dimensional disaster subject positions primarily around the axes of gender, caste and age. These categories however fall short of capturing the complexities of lived experiences of inequalities and often further marginalize the most vulnerable by excluding them as ineligible or undeserving victims of disaster interventions [11]. Thus, in an act of co-opting inclusion, the formal actors contribute to creating categories of deserving and undeserving beneficiaries [11]. As these processes ignore the role of structural violence in shaping the differential consequences of disasters faced by the marginalized [25] it results in the most marginalized groups being excluded as the undeserving category. It is in this context that the current paper attempts to revisit issues of structural inequality and justice in disaster recovery from the perspective of the most marginalized communities faced with recurrent disasters in the Indian context.

## 2 Theoretical framework

The two forms of justice that are key in the debate concerning social justice, in the contemporary world of increasing frequency and intensity of disasters, are distributive and procedural justice [26]. In the disaster context, distributive justice is concerned with the division of benefits and the allocation of burdens, in the particularly difficult scenario of scarce resources and competing claims and needs [12]. Procedural justice on the other hand is linked intimately with issues of participation and stakeholder engagement [27]. In the case of procedural justice, the process is the only criterion for a just outcome [50]. Distributive justice is the harder of the two concepts to materialize and a fundamental reason for that is the question of ‘equality of what’ since most normative theories on social justice tout the need for equality [[46], [50]].

Peraccini [28] problematizes the preoccupation of theories of justice on “how” and “what” can be distributed, instead of focusing on understanding how an unjust distribution really takes place and the constraints which weaken the institutions of distributive justice. To be able to make this shift proponents of ontology of possibility or potentiality push for a change in gaze from formal actors and institutions to that of lived experiences of marginalized subjects at the local and every day scale [[29], [30], [31]]. These scholars believe in the potentiality or possibility of learning from subversive and radical practices and thus overcome the hopelessness created by the critical literature regarding the unchallenged hegemony of neoliberal government rationalities and stiffening of alternatives. Hence we draw from scholars outside the discipline of disaster studies who focus on issues of structural inequality and injustice from the critical lens of the politics of neo liberalism [3,4,19,20,32,33] and that of the ontology of possibility and potentiality [[29], [30], [31]] to understand lived experiences of structural inequality and injustice and the community's initiatives to address it in the post disaster recovery context. Spaces beyond the public arena where everyday social relations and processes that create categorical social positions, privileging one over the other play out, are key to understanding and addressing structural inequality and injustice [20]. Thus, drawing from proponents of ontology of possibility [30], we turn our gaze towards subversive or radical practices in the post disaster recovery context that illuminates the everyday social relations and processes and its interface with formal actors and institutions, in addressing the issue of complex structural inequalities and injustice, and the challenges of externalizing rights claims.

## 3 Objectives and methodology

By drawing from the above literature and theoretical framework the paper proposes to explore: The lived experiences of structural inequality and injustice among marginalized communities; the factors contributing to the persistence of injustice; the community's concept of justice; and its assertion and the challenges it encounters to operationalize justice in the post-disaster recovery context. The study uses the multi-sited instrumental case study approach to study the phenomenon of structural inequality and injustice in the context of disaster recovery. The approach is best suited for a qualitative study that is exploring new theoretical possibilities to understand how injustice could be approached in disaster recovery. The sampling strategy of intensity sampling was used to sample two information rich cases that manifest the phenomenon of interest intensely. Case studies of movements by marginalized communities living with disasters were purposefully chosen for the given study. Thus, the two case studies (Thayillam and Vistapit Mukti Vahini) are chosen as a comparative point across other cases in the Indian context in which the phenomenon (community assertions to address structural inequality and injustice in the context of disaster recovery) might be present. The principle of maximum variation was also used in the short listing of case studies to represent varied and contrasting context of institutional response to issues of structural inequality and its implications for the most marginalized in the post disaster recovery context. Kerala was hailed as a successful case study of land reforms, whereas Bihar was the worst. On similar lines the two states contrasted each other with regard to their performance in various growth and development indicators such as the Human Development Index, literacy rates, gender ratio etc. As disaster management is a state subject the selection of two case studies also introduced different disaster profiles, policy and institutional context with regard to disaster recovery as well. Thus, the two case studies incorporate a wide range of dimensions that shape social relations and processes that contribute to the persistence of the issue of structural inequality and injustice in the post-disaster recovery context. The commonalities that emerge out of these contradictions enable us to pick up key themes to understand the phenomenon of interest.

**Fig. 1:**
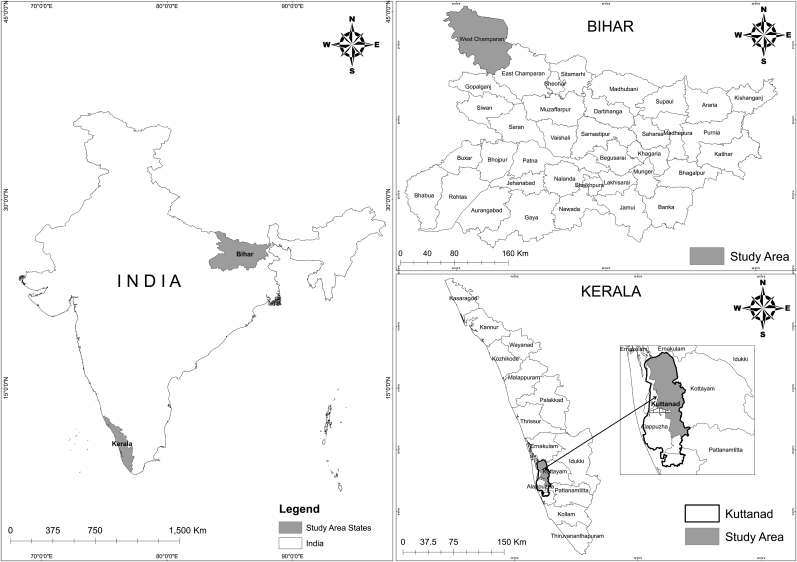
Map of study areas.

The use of the case study approach facilitates the use of multiple data sources, and for the current study we have used a range of methods starting with observation of three public events, nine in-depth key informant interviews with leaders of the two initiatives, four focused group discussion with the leaders and members of the collective, two audio-visual material, two live performances of art forms such as songs and dance, and other secondary sources of data such as pamphlets, notices and publications emerging from the movement. The data emerging from various sources was transcribed and synthesized to present a holistic understanding of the community assertion and the context in which it is located. Fig. 1 illustrates the study areas that were part of this study.

The analysis below starts with the case study summaries of the two community assertions against structural inequality and injustice in the disaster recovery context. Further the analysis section captures the key themes emerging from the two case studies illustrating the understanding of: structural inequalities and injustice in the context of disaster recovery; and the alternate conception of justice and disaster recovery emerging from among marginalized communities living with recurrent disasters in India. Our analysis illuminates how disaster risk and recovery emerge from historical and everyday lived reality of marginalized communities, their social relations and resulting material conditions. And that the challenging of everyday social relations, inequality and injustices is central to the community's alternate conception and assertion for disaster recovery. We propose a conception of disaster recovery that is not state centric, but as an ongoing process of challenging everyday social relations, inequality and injustices that the communities have historically encountered, primarily at the local and everyday scale. All the interviews and FGDs were conducted in vernacular languages and dialects, and direct quotes used in this paper have been translated by the authors into English.

*Vistapit Mukti Vahini*:The Vistapit Mukti/Sangharsh (struggle) Vahini is a movement by the people who have lost their homes and livelihoods due to *katav* (riverbank erosion) in Bihar. The landless and marginalized were disproportionately affected by the *katav* as those with means, specifically landowners, were able to relocate to better areas and retain landownership, while the marginalized who lacked both homestead and agricultural lands were left to live in dangerous areas like roadside embankments.

As a witness to this somber reality, Bijay Manjhi, who belongs to a Maha Dalit community, the Musahars, was displaced as an infant due to *katav* in 1977 and forced to live at a roadside embankment in Bankatwa, began the struggle in 2002. Manjhi, who pursued a postgraduate degree was forced to drop out due to financial constraints, started the struggle after realizing that basic needs and rights were being denied to his community. His first act, at the age of 25, was writing a formal application in February 2002 to the Circle Officer in Nautan Block (West Champaran District), requesting rehabilitation of 53 families in Bankatwa. When Manjhi refused to accept the bribes offered by landlords, he was threatened and implicated with false First Information Reports (FIRs) to deter him from this movement. But he persevered despite it all and gained the communities support for the struggle. This allowed the struggle to gain increased membership and evolve into a grassroots level micro movement.

In the initial stages of the struggle in 2002, membership was open to any community displaced by the *katav*. Later, about two to three years after the struggle began; it was restricted to a collective few who belonged to the most marginalized sections. The divisions within the larger group were mainly due to conflict of interest and misappropriation of the struggle by the landlords (the dominant groups who at that time were also part of the struggle). Presently, the struggle is left with the most marginalized from among the *katav* displaced population. The struggle, therefore, consists mostly of ‘displaced landless’ individuals and families. The struggle soon opened its doors to people affected in other ways i.e. not just *katav*, resulting in members from other villages of various marginalized communities and religions joining in the struggle.

*Thayillam Collective*: In 2006, the historically marginalized landless community in Kallara formed a collective known as Thayillam, to reclaim flood prone fallow land for sustenance farming. This movement was inspired by the success of the Tribal movement led by Adivasi Gothra Maha Sabha in front of Kerala State Secretariat in 2001. This tribal movement was started to gain farming land for the Adivasi community to enact a community farming initiative.

Mr. Thankachan C. J., who was part of Adivasi Gothra Maha Sabha in the community farming initiative brought this concept into his community and the village he belonged to in Kallara. He belonged to the landless farmers’ class that also does inland fishing.

The Thayillam collective was a response to the exclusion of Dalit from Kerala's land reforms. Thayillam proposed to continue farming and the community decided to become farmers rather than live and die as landless agricultural workers.

It was not a simple decision based on economic rationale alone but came across as an ancestral wish. The collective believes that the land is that of their ancestors who had sacrificed their lives for it, and it's the posterity's responsibility to carry forward the struggle. In one of the annual rituals performed to commemorate the spirits of the ancestors, the community encountered the unrest of the ancestral spirits for having deserted them and their land. Thus, the Thayillam collective decided to take forward the learnings from the community-based farming initiative of Adivasi Gothra Maha Sabha.

The Panchayat was willing to cooperate with the collective as they wanted to get rid of fallow land owned by locals and people from other districts. The collective was also supported by the Farmer's Association and was able to successfully reclaim land for cultivation. The movement got wider local support and they could lease about 142 acres of paddy field for cultivation. Their farming practices are deeply connected with the local wet land ecology and is a sustainable method of farming. Their sustainable farming practices emerge from their traditional knowledge of the local ecology. Thus, they do not see floods as disasters but as very vital natural processes critical for their local ecosystem dependent way of life and well-being.

The community believes that the local environment forms the crux of their individual and community identity. Thus, the decisions that affected the environment that they live in and depend on for livelihood should be made by the local people and not by external entities such as the state that served to only destroy their environment as well as their well-being.

## 4 Lived experiences of structural inequality, injustice and community assertion in the context of disaster recovery

Drawing from the critical literature on theories of justice and politics of neoliberalism, the sub-sections below elaborate the two case studies from five analytical perspectives towards understanding how an unjust distribution really takes place in the disaster recovery context. Firstly the analysis attempts to illuminate how the marginalized communities have been historically failed by development, welfare and inclusion projects; secondly, how these complex inequalities and injustices transition into lived experiences of disaster risk and recovery among the most marginalized sections; thirdly, how the marginalized community's historical socio-political context shape the community assertions differently; fourthly, how the marginalized communities understand the manifestation of structural inequalities and injustices and assert themselves in the disaster recovery context; and finally, how disaster recovery is conceptualized (as an ongoing process of challenging everyday social relations, inequalities and injustices) at the local and everyday scale. The discussion and conclusion sections bring forth the alternate theoretical perspectives on disaster recover emerging from the in-depth analysis of the two case studies.

### 4.1 Historical socio-political factors contributing to the persistence of structural inequality and injustice in the disaster recovery context

Structural inequalities emerge because of several factors converging together to leave certain communities extremely marginalized. This section explores the historical socio-political factors that contribute to persistence and worsening of structural inequality and injustice in the disaster recovery context:The social structures of caste system that existed in India especially within the agriculture sector; the colonial idea of development and its imprints on post-independence era; and the failed welfare and inclusion projects of independent India. The next sub-section draws out the interface between these failures and that of the differential distribution of disaster risk and recovery experiences.

#### 4.1.1 The caste system in India

The pre-independence social structures of caste system determined the role of land owners and laborers in the agricultural sector. The low status groups such as the Pulayas in Kuttanad [21] and Musahar in Bihar, among other scheduled castes were the slaves of the higher caste. After the abolition of slavery by royal decree due to British pressure in 1855, the relationship shifted to one of attached labor (ibid) or bonded labor which still persists in the state of Bihar [34]. Thus, the system of laborers and land owners was not determined by choice or economic constraints but rather by the social stratification that existed in the two states across time, and continues to persist till date as the development, welfare and inclusion projects have failed to address these inequalities and injustices.

#### 4.1.2 The Skewed Idea of development

The colonial idea of development was largely a project of securing metropolis capital in peripheries [52] that left its imprints on development planning in post-colonial countries. Unequal distribution of the benefits of infrastructure development and the dominant group's ownership of infrastructures are the imprints of colonial influences in India's development. The skewed idea of development emerging from the colonial thought process was also evident in the agriculture sector to the detriment of marginalized communities in Bihar and Kerala.

The British Empire established the Zamindari system in India which resulted in land owners gaining official recognition and being compelled to pay land revenue. For the sake of tax payments, the Zamindars in turn squeezed the labourers dry. Commercial agriculture became the norm as the British required primary produce such as rubber, coffee, indigo etc., to boost their industrial production. Across the states of India, including Kerala, this resulted in people having to change the type of crops being planted from food crops to cash crops [35]. The reclamation of wetlands in Kuttanad and the irrigation projects in Bihar that continued well beyond the colonial period to ensure food security of the growing population of independent India, too had significant negative implications for environmental and social justice The reclamation of wetlands which started from 1865 with the Pattom Proclamation by the ruler of Tiruvitamkoor Kingdom [36], intensified with the privatization of land ownership through the transfer of land from the temple collectives to the tenants (ibid). Since 1947 (Independence) the economic, environmental and social technologies used to altering the land use and land cover patterns went through drastic changes (ibid). The construction of Barrage, irrigation channels, canals, bunds and sluices changed the water spread and ecology of the region substantially. The most impactful of these constructions was the Thannermukkam Barrage in Kuttanad and Gandak Barrage in Bihar.

The Thanneermukkom barrage effectively divided the ecological zone of Kuttanad from a single zone of brackish water, into two distinct agro-ecological zones, with one being a saline zone and the other a zone of controlled salinity [37]. This along with agricultural practices such as land filling, double cropping of paddy, cultivation of perennial/cash crops like coconut instead of paddy, leveling of water spread areas and encroachment were all characteristic of this period [36]. All this was done to extract the maximum economic profit from the land reclaimed in the previous era.

The Gandak barrage in Bihar, serves as an example of the skewed idea of development, to the detriment of marginalized communities. The Gandak is a Himalayan river that flows through Nepal to India. The construction of the Gandak barrage (head works and canals) had been envisioned as early as the 1860s in the wake of the famines of 1865–66 [38]. The British machinery in India dropped the proposal owing to competing demands in other regions (Tirhut and Saran Divisions in Bihar) and the negative ecological implications of land degradation due to salt efflorescence (ibid). Though the second proposal in 1871 was also rejected based on similar reasons as the first, work in the form of Saran canal were executed and completed in 1881. A famine in 1896 prompted another dream for the use of Gandak's waters for irrigation. As a by-product of famine relief work, the Tribeni canal was constructed by the British in 1914 to channelize the water of the river Gandak.

Post-independence, to boost food security the Gandak project was once again proposed to address the irrigation requirements of districts in Bihar, Uttar Pradesh and Terrai region of Nepal [38]. Though the barrage and related infrastructure of canals, irrigation channels and embankments for the purposes of river training were planned, there were significant challenges in the execution of the project and the project is yet to be completed and realize the full potential of the promised benefits.

#### 4.1.3 Failure of welfare and inclusion projects

The recognition of disparities and inequalities in the new independent India, the agrarian unrest and demand for land distribution led to several welfare and inclusion projects. However, these projects failed to address the pre-existing structural inequalities and worsened the disparities. For example, the movement of land reforms in India were started in 1960 and many state governments initiated the process [35]. However, even after six decades, land reform in India is still an incomplete project. The motto of ‘land to the tillers’ is yet to be fulfilled in the country. The landless agricultural laborers from the Dalit communities in Bihar and Kerala are the direct victims of the failed land reforms movement [39,40], that failed to address the root causes of pre-existing structural inequalities and further aggravated the travails of the most marginalized.

The land reform movement in the state of Bihar was initiated in 1950 with the Land Reform Act that brought an end to private ownership of several kinds of non-land immovable properties [39]. This was followed by the 1955 Bihar Agricultural Land (Ceiling and Management) Act for preventing excessive private ownership of land, and it was successfully stalled by resisting landlords (ibid). Finally, in 1961 the Land Reform (Ceiling, Land Allocation and Surplus Land Acquisition) Act came into effect in the State and was further revised in 1971 and 1973. The revised versions of the act had several provisions that enabled the maintenance of the status co and did not help in addressing the issue of landlessness emerging from caste based structural inequalities and injustices, as evident in the statistics of landlessness in Bihar. According to the 55th round of NSSO's survey (1999–2000), of all the agricultural labourers in Bihar nearly 76.6 % are completely landless (ibid). According to a survey by the Department of Revenue and Land Reforms, Govt. of Bihar, a total of entitled 2, 16, 829 homeless Mahadalit families were identified. According to VMV statistics at present there are at least 35,000 katav displaced population in West Champaran, and most of them belong to socially marginalized caste groups. Though, Kerala is hailed as a model state for land reform, the plight of the landless agricultural laborers from marginalized communities was no different from Bihar. The agrarian unrest led by communist ideology demanded for land reforms and in 1957 efforts to enact land reform was initiated by the newly elected communist party of India [40]. After a decade long struggle the land reform was finally enacted, but contrary to the original formulation, the enacted act granted cultivable land to tenants and not the landless agricultural laborer [21,40]. The landless who tilled the wetlands were given homestead land, minimum housing and menial government jobs. Thus, their identities changed from landless agricultural laborers to that of daily wage laborers. The agriculture reforms that followed the land reforms too favored large landowners engaged in commercial agriculture. Thus, the agricultural reform continued to follow the British era agricultural development model, to the detriment of marginal sustenance farmers [35].

### 4.2 Manifestation of historical socio-political failures as differential distribution of disaster risk and recovery

The analysis of the two case studies illustrates how historical structural inequalities and injustices manifest as differential disaster risk and recovery experiences among the marginalized sections of the society through three predominant pathways. Firstly, the marginalized sections of the society are forced to occupy physical locations that are more prone to disaster risk and thus experience recurrent disasters as in the case of Bihar and Kuttanad. Secondly, disasters often become a catalyst for the initiation of development and welfare programmes as part of disaster recovery interventions which further exacerbates injustice due to the neglect of pre-existing structural inequalities. Finally, disasters compound these inequalities further as they just do not strike a physical space but also impact the socio-political structures of the society that further aggravates inequalities in the disaster recovery context.

The skewed idea of development inherited from the colonial past resulted in the disruption of the ecological balance and social relations between the inland fishing and agriculture communities in Kuttanad. The floods that replenished the wetland ecology, and the social relations between communities were critical for the livelihood sustenance and food security of the marginalized groups (Thayillam member). The inland fishing community required the paddy fields for fish breeding and this was possible when paddy cultivation was seasonal (but not anymore), while the agriculture community would use organic waste and matter from the fish as nutrients for their land. The natural cycle of flooding that replenished the fields with nutrients and fish seedlings, was either disrupted by development projects or brought in contaminants from the increasing urban sprawls and the inorganic double cropping of paddy and other cash crops [37]. This further damaged the symbiotic relationship between the fishing and farming communities resulting in the disruption of traditional livelihood practices and food security. In the context of double cropping of paddy and cash crops, floods are no more beneficial and bring in heavy losses making agriculture an unviable occupation in the wetlands. This has significantly affected the proportion of land under agriculture, resulting in fallow land or reclamation of land for cash crops/non-agricultural purposes (Thayillam member). The profit-loss calculus of agricultural reform also required that land be left fallow to prevent burdening the land and depriving it of its ability to sustain cash crop production. Leaving land fallow or converting it for non-agricultural purposes too had significant negative consequences for the wetland ecology and the livelihood of the landless laborers who were now dependent on agricultural wage labour. Historically, institutional mechanisms have ignored the plight of wetland ecology and these landless laborers while addressing the issue of wetland reclamation for non-agricultural purposes (Thayillam member). For instance, even the recent Kerala Conservation of Paddy Land and Wetland Act, 2008 restrict the conversion of paddy field for non-farm purposes including reclamation. However, it does not prevent anyone from leaving their land barren. Thus, this Act does not address the concerns of the wetland ecology and landless community who depend on these lands for their livelihood. This led to the destruction of: the bio-diversity of the ecosystem; the traditional agricultural practices; and the wellbeing of the most marginalized communities as their source of sustenance was disrupted, pushing them into menial jobs and wage labour. The destruction of the ecosystem and the bio-diversity had implications for the life style of the most marginalized especially regarding the change in their food baskets, which could no more depend on the organic produce from the immediate wetland ecosystem. The technocratic approach to agriculture and alienation of traditional practices, along with the welfare schemes for creating housing colonies for landless laborers and providing menial jobs as their source of sustenance, has had a detrimental effect on the identity and dignity of the community (Thankachan). Often the colonies were situated in areas that were regularly flooded, however recurrent floods that these communities encountered were never considered as a disaster warranting non-routine interventions.

In the context of Bihar, the construction of the barrage and embankments to contain the river and enhance irrigation potential resulted in communities on the Indian side having to relocate [41]. Marginalized landless communities were left behind as there was no legal provision to relocate them and they had to face new challenges as the barrage now changed the nature of flooding. With the construction of embankments to contain the flow of the river, the regular floods became more unpredictable, intense and deposited more silt on the river beds leading to riverbank erosion and sudden change in river course [41]. Water scarcity during summer and flash floods due to breach of embankments were also among the other issues that the communities had to face. Though the full potential of the project is yet to be realized, the negative consequences especially for the marginalized sections far outweighs the benefits.

The river Gandak is the most notorious with regard to riverbank erosion. It is responsible for leaving families that call its banks home – homeless and landless. The families that are affected by *katav* are left only with the option of relocating outside the embankments. However, the support extended by the government for rehabilitation is often unavailable, to the most marginalized landless communities as they did not own land that could be compensated. Hence, they continue to live within the embankments encountering floods on a regular basis. With no proper rehabilitation after the Katav, some of the displaced groups settle down on embankments by the roadsides [42]. This further increases their vulnerabilities since the embankments are at risk of further erosion. The landed communities who had relocated outside the embankments with the support of government compensation, retained their land ownership and cultivated the same with the help of landless laborers who had no option to leave the flood and *katav* prone areas [42].

The extension of an idea of disaster assistance and recovery that neglected pre-existing inequalities, very similar to the idea of development and welfare efforts described in the previous section, contributed to the persistence of structural inequalities in the disaster recovery context. The minimalistic emergency and event centric disaster assistance or recovery efforts ignored the root causes of pre-existing structural inequalities and thus exacerbated the vulnerability rather than addressing it. In most instances the communities displaced by disasters were left to fend for themselves, with only minimal support in the form of immediate rescue or relief services such as dry rations, tarpaulins etc. Most of these services would not even reach the marginalized communities due to the complex social relations and processes that play out in these contexts. Welfare programmes such as the ‘Indira Awas Yojana’ were extended to support the building of concrete homes in the disaster recovery context, however these programmes were largely applicable to those who owned homestead land and no programme addressed issues of landlessness. In instances when the welfare homes were destroyed by *katav*, the beneficiaries were not eligible for a second house and were often threatened of being subjected to legal action upon not completing the construction of the homes that were swept away by the *katav* and floods.

Yet another factor contributing to the persistence of structural inequalities in disaster recovery, is the complex social relations and processes that play out in the everyday context that exploit the marginal status of the most vulnerable groups and the services targeting them. In Bihar the low educational status of the community and the historical practice of caste-based discrimination and untouchability is upheld even today to exploit the most marginalized sections. The development and welfare programmes are an opportunity for illicit income for all dominant stakeholders in the given context. It is highly prevalent at all levels of the society starting with: landlords who use their cheap bonded labour in return for a place to stay [42]; the government boat man who extracts exorbitant cost for ferrying communities across the rivers especially during disaster times [43]; the local government officials and functionaries who have to play a key role in establishing the identity and eligibility of these communities for welfare programmes and other basic entitlements; the engineers who are entrusted with the task of embankment strengthening; the functionaries and middle men at the block and district level who manage a cut from every welfare benefit that is extended to the marginal communities and finally the local and state political actors who see the community only as a potential vote bank. The communities believe that katavs are mostly due to the laxity of the government and its bad decisions. The ‘expert’ knowledge of the engineers who were brought in by the government is attributed to the disturbed flow of the river. This has led to recurrent episodes of katav in the region and the locals expresses their concerns regarding the same as follows:

“See, the government does not include the people who are born and raised here in the decision-making process … Whenever we experience a flood, that's when we see the engineers. One time when we faced floods in 2003, the engineers described the river as ‘Lakshminiyaan’(The Goddess of Money)– because they earn through the floods. 5 or 10 houses were taken away by katav, but their attitude didn't change even then. So, we asked them to take the suggestions from the locals. But they rejected the idea completely saying that they are highly educated and don't need suggestions from us.” respondent from Godeya village, Bihar.

The mainstream conception of disaster recovery does not go beyond the event centric approach of emergency relief and rebuilding, to address pre-existing structural inequalities, and thus does not address the concerns of the most marginalized.

### 4.3 Past social movements and disasters as catalyst for community Assertion Against Structural Inequalities and Injustice in the disaster recovery context

This subsection deals with the two movement's linkages to past social movements and recurrent disasters in the given local context. It also illustrates the impact disasters can have on their struggles either serving as a catalyst for the struggle or as a new factor that is to be incorporated into their struggles in order to achieve a better form of resilience.

#### 4.3.1 The historical movements that set the stage for the two initiatives

The two communities' assertion for justice are shaped not just by the historical context of oppression, but also by the rights and identity claims of the past within the respective contexts. For example, Champaran is the region from where Gandhi's non-political grassroots struggle for the cause of poor and exploited peasants, the ‘Champaran Satyagraha’, started in 1917. Jaiprakash Narayan's movement demanding a total transformation or freedom from all forms of oppression and injustice dominating the landscape of independent India in 1974 was also launched from West Champaran. The Vistapit Mukti Vahini was influenced by these movements in terms of ideology, which is based on Satyagraha and Peaceful Total Revolution, committed to non-violence at its core. According to Pankaj (lead activist), the struggle imbibed the philosophies of Buddha, Ambedkar, Gandhi, Jesus, and Sukrat. Pankaj and Manji associated with the movement since its initial days, describe their peaceful assertion as follows:

“The villagers started gathering outside the Block Office. They included both men and women. We were at least 150 people from different villages: Mangalpur, Sivrajpur, etc. We did not actually lock the office. We just occupied the office space and stopped all work. We did not allow any official to work. The name tala bandi is just a symbolic act. As the blockade continued, the police arrived in a force of 100. The BDO was not in the office that day. But the CO was. So, we sat in the Circle Officer's room and did not allow him to come out. The CO was quite tensed. You must understand that we do such things out of desperation. At that time our movement was not widespread. To ensure that there is no violence, we also put women in the room. Of the six there, four were women who made sure that the CO was not harmed. Outside, other members argued with the officials over the delay in getting the applications for rehabilitation processed. Somehow, the police managed to disperse us”.

The Thayillam Collective was started by members who had been part of and witnessed struggles by several marginalized groups such as that of the Dalits and indigenous people in Kerala. This allowed them to understand the harsh realities that the marginalized groups faced and very often these struggles resulted in them perishing under oppressive conditions. The struggles by these oppressed groups that enlightened the members include: the successful 11 K.V line struggle that had a Dalit community opposing a project to lay a high-tension electricity line in 1995: the 1996 indigenous people struggle to get back the land that they had lost due to threat, loot and duping by aggressive settler cultivators in the 1930s; the Muthanga Struggle where police firing had happened for the first time ever in Kerala against an Adivasi struggle; the struggle by indigenous groups in Aaralam, Kannur District, to gain the land that had been earmarked for them and many more. These struggles formed the foundation of the faith for the Dalit community in Kallara to launch their own struggle, since 2006, which would expand to include around 500 families for claiming flood prone fallow lands for sustenance agriculture and environmental conservation.

Mr. Thankachan C. J., one of the progenitors of the struggle had the following to say:

“I would say that the Dalits in our area lost sense of community and belongingness through encounter with forces of sectarian politics, religion and so on. Our activity is an effort to reclaim the community and its identity that is scattered in different ways. Thus, the physical and the socio-cultural reclamation are synergistic.”

Thus, these assertions cannot be isolated as standalone rights claims or disaster risk reduction or recovery effort, but to be understood as an integral part of their identity and way of life. Though both groups belong to the most marginalized Dalit communities, they do not use the Dalit identity for mobilization of vulnerability. In the case of VMV the subject position of “disaster displaced” is the prominent identity around which they mobilize, probably as a way of rejecting the cultural and historical impressions that the Dalit identity elicits in the context of Bihar. Though the Thayillam collective mobilizes the identity of a farming community, it stays strongly connected to its Dalit identity and the pain of historical and cultural oppression through its art performances such as folk songs and dance. They practice rituals contrary to organized Hindu religion in the state and they also conduct series of study groups called Adhishakthi Padashala to promote their way of farming and their approach to ecology. They conduct local learning centres for children to learn about their past and all these initiatives are primarily implemented through women workers.

#### 4.3.2 Disasters as catalysts for the assertion against structural inequalities and injustice

Both communities were familiar with floods historically however with the advent of large-scale development projects for the promotion of agriculture as a predominant economic activity, the intensity and the nature of disasters changed dramatically. The beneficial floods which was essential for the replenishing of land and inland water bodies started becoming destructive and devastating. The floods no longer replenished the wet lands of Kuttanad but degraded the biodiversity of water bodies. Thus, it had negative implications for sustenance and well-being, including the nutrition and health, of the marginalized communities who lived in these flood prone regions. In Bihar it changed the river course and ferociously eroded the river banks of Gandak that housed the marginalized landless laborers on the outskirts of the village, including the few homes provided to the welfare subject on Government land.

Disasters played a critical role in the intensification of pre-existing pain and suffering emerging from the everyday experiences of structural inequalities and injustices and thus became a catalyst for the initiation of the VMV. The experiences of Thayillam collective also demonstrates how they have integrated floods into their everyday lives and livelihoods that are resilient to disasters and opens newer conceptualization of disaster recovery in relation to addressing pre-disaster conditions of structural inequalities and injustice. The members of the collective recall their recent experiences of flooding in the state of Kerala as follows:

“We live with floods every year. We have nothing much to lose from the yearly floods. Our belongings are minimal, and we know how to swim to reach safe locations as it has been an everyday reality for many of us while we were growing up. We had to swim to reach our schools or any other facility beyond our hamlet. Every year when our fields and homes were flooded no one bothered. But this year when the entire state was flooded (2018), we too had people coming forward to put up relief camps and reaching out with supplies. We can accommodate many floods as we do not engage in capital intensive agriculture and hence our losses are not catastrophic enough to deprive us. In fact, we want our fields to be flooded every year so that it can bring the fish and the seedlings for the aqua culture.”

Though disasters become catalyst to the community's assertion, they are seldom conceptualized from an event centric perspective. The assertions of both the communities are very much part of their everyday lives, against the root causes of differential disaster risk and recovery outcomes, among the most marginalized sections. The community whom the movement represent are the victims of failed developmental projects in both the states. The community assertions emerge out of multiple failed encounters with government interventions and stakeholders which control and seldom recognize community's perspective. To be able to address the material conditions of vulnerability (landlessness), identified to be the root cause of their vulnerability in general which includes differential disaster risk and recovery outcomes, the movement had to conduct protest march and non-compromising engagement with the agricultural department, revenue department and most importantly the local paddy land owners who wish to keep the land barren rather than give it on lease for farming. Through lease farming, the Collective could build a farming practice and a farming community in the locality. The Act and the lease farming would indeed prevent conversion of these paddy fields in the near future, and preserve the wetland ecosystem which is integral for livelihood and ecological resilience in the context of disasters. The collective could also successfully combat the neo-liberal land use policy and capitalizing local ecology. Through continuous protest and engagement with the revenue and disaster management authority in Kerala, the Collective could ensure that disaster compensations were given to the lease farmers and not to the land owners. The collective had to conduct a protest march and series of protest movements in front of the district administration office to convince the government that the lease farmers are the cultivators who need to be compensated following the 2018 floods and not the land owners. It was perhaps for the first time in the country that flood affected lease farmers could pull together and raise voice against the conventional method of disaster compensation. This is indeed a first step towards institutionalizing this new method of disaster compensation. On similar lines the VMV that began as a single individual writing application to rehabilitate 52 families has now become a movement of 30 communities displaced by river bank erosion. They have managed to settle 562 families and capture 130 acres of land for the purpose of rehabilitation of displaced communities.

### 4.4 Community's alternate conception and assertion for justice in the disaster recovery context

This, sub-sections attempt to capture the essence of the alternate conception of justice, the drive and the convictions that keep the movement alive. The alternate conception of justice built around the synergy between the cultural belief of the community, environment and human well-being, identifies landlessness emerging from historical and cultural oppression, as the root cause of marginalization and attempts to address the same. Their drive can be located within their need to alleviate themselves of their material conditions of vulnerability and their convictions are rooted in the alternate conceptions of justice and their everyday strategies of challenging inequalities and injustices. They have been able to understand the limitations of the neo-liberal agenda and their place within the state's plans allowing them to formulate plans towards their own ideal of a better life with or without the state's aid.

#### 4.4.1 Alternate conception of justice

The approximate translation of “Vistapit Mukti Vahini” is “The Liberation Movement of the Displaced”. Liberation from historical and cultural oppression, by addressing landlessness - the root cause of marginalization, has been the concept of justice in VMV. The rejection of empowerment and embracing of liberation underline the recognition of oppression (and not backwardness) as the starting point of addressing issues of structural inequality and injustice. Though the movement draws from the ideologies of historical movements in the context and the guidance and support of other ongoing movements such as the “Parchadhari Sangarsh Vahini” and “Lok Sangharsh Samiti”, it has organically emerged from within the community of displaced and is fully supported by the contribution of each member as described below:

“All three struggles are friends of each other. They participate in each other's struggle. They unite as and when required, which is how they derive their strength. They function separately but come together as and when required. For example, if there is a program / strike called for the Vistapit, every individual participates at his/her own cost. But the expenses incurred on reaching the venue, all other costs like food and refreshments will be covered by the members of Vistapit. For the funding part, it was decided that every household will contribute ₹5 if the event is at the Block level, ₹10-₹20 for Zilla and District headquarters, and at least ₹50-₹100 for events organized at the state level”.

The Thayillam collectives' conception of justice is built around the synergy between the cultural belief of the community, environment and human well-being. The cultural and spiritual beliefs of the members regarding the significance of the spirits of their ancestors and that of the land are an important component of the conception of wellbeing and justice. The community believes that they cannot achieve wellbeing if the spirits of the ancestors and that of the land are not taken care of. Leaving the land fallow and fragmentation of the community caused significant distress to the spirits and the same was revealed to them through the ritual called “Undhan” during which they invoke the spirits of their ancestors to seek their blessings and also get to interface with them. It is based on this realization that the Thayillam Collective was formed to reclaim the land that housed their ancestors' spirits and ensure its well-being, which in turn was intrinsically connected to environment and human wellbeing. The Thayillam Collective's conception of justice is that of synergy between Spirits, Environment and Humans, leading to comprehensive well-being that is also sustainable.

#### 4.4.2 The need to alleviate the material conditions of vulnerability – landlessness

Both the community initiatives locate itself within the discourse of material conditions of vulnerability – specifically of landlessness and the historical failure of the state in addressing the same. The initiators of the movement articulate the same as follows:

“Everyone needs land and a voice that can be heard. It was haunting me for quite some time. Hence, I decided to start the struggle (Manji)”

“Our identities were tied to the land that we tilled. Without our land we were reduced to mere unskilled labourers. Our rich knowledge of cultivating the wetlands gained historically was discounted along with our dignity. Reclaiming agriculture land and our agricultural practices is also about reclaiming our identity and dignity (Thankachan)”

However, the two communities frame the initiatives very differently. Though the VMV recognizes the limitations of depending on the state and the subject position of the disaster displaced, their rights claim relied on the 1991 resolution of the Relief and Rehabilitation department, Government of Bihar, to provide 4 decimal land as homesteads to the katav displaced. Recognizing the limited potential of rights claim and political action around such subject positions Pankaj shares: “Circulars are not legally binding. No legal action can be taken against the officers responsible. Therefore, legislation is necessary. Unlike circulars, Acts are enforceable”. The Thayillam Collective on the contrary subversively used the state's policies and programmes to achieve their purpose of addressing issues of structural inequality and justice, which was very different from the local government priority of promoting agriculture on fallow land. Thus, the local government supported the Thayillam's initiative to acquire their ancestral land on lease from those landowners who had left it fallow. The members of the Thayillam collective were owners of homestead land provided to the landless labourers, and the house provided by the welfare programmes that followed. Some of them even had menial government jobs of sanitary workers and sweepers. However, in-spite of being beneficiaries of various welfare programmes they struggled with the pain of indignity and estrangement from the very land and ancestral spirits that constituted their identity.

The two-initiative's disparate starting points could be attributed to the contrasting context of structural inequalities in the two states, their differential outcomes with land reforms and the nature of disasters and its implication. However even amidst these differences we can see commonalities in the struggles of the communities with disasters, the exacerbation of pre-existing structural inequalities, the alternate conception of justice and the strategies for addressing the same.

Strategies for Addressing Structural Inequality and Subversively Negotiating the Neoliberal Politics: Both communities recognize the fall out of a political society in place rather than the much-preferred civil society of rights bearing citizens. Thus, in Bihar, the VMV members negotiate with the so-called political actors through their subversive practices as follows: “When the time came for the next election (November 2005), all the candidates were called for a Sabha (meeting) in Betiah, Champaran District Head Quarters, Bihar. They were made to write their promises. The promise of 4 decimals of land for each of the displaced read, ‘I will get the work done in two years if my party forms the government. I will resign from the position of the member of Lok Sabha if the work is not done within two years’ and signed by the candidate. Using these written promises VMV has managed to demand and secure resignation of political actors who did not deliver on their promise and gained momentum for the process of resettlement.”

On similar lines the Thayillam collective recognizes the politics of neo-liberal welfare extended in the form of subsidies for pesticides, inorganic fertilizers and hybrid seeds, pushing a form of agriculture, which they recognize as unsustainable and as the root cause for the marginalization of the farming community. Thus, they rejected the formal institutions, the universal technocratic agriculture knowledge of formal actors and the subsidies that contributed to unsustainable practices. They relied on the community's traditional knowledge to reclaim their bio-diversity, well-being and dignity through organic farming and other traditional agriculture practices of alternating paddy cultivation with aqua culture, preserving local seeds, shifting to cultivation of millets, among others. This has made agriculture less capital intensive and free of catastrophic risk faced during floods and disasters, as expressed in the following narrative: “The agriculture is managed primarily by the women, as they have been the guardians of traditional agricultural practices geared towards sustenance and well-being. Men look for quick profit and thus easily turn towards unsustainable practices, with economic gains as the only outcome prioritized. By going back to our traditional agricultural practices our biodiversity has been revived, our food baskets have changed to include locally produced fish and other nutritious tuber and leafy vegetables that preserved our health and wellness in the past. Now all of us in the collective have become home and landowner with livestock, from that of landless lease farmers. Thus, through our every day practices we demonstrate that sustenance-based agriculture is viable for the farmer, not just in economic terms but in ensuring holistic wellbeing” – Treasurer of the Collective.

The VMV uses several non-violent radical strategies of writing applications, fasting, protest, creating blockades, picketing, pressure tactics, and contracts with potential political candidates, among others. The very act of being part of the movement is understood to be a process of bringing about change in psychic resistance to oppression and marginalization as expressed by the members: “Through the struggle, people become aware of their rights and entitlements which would not have been possible otherwise. For example, it was only during the struggle that they came to know that 4 decimal land is their entitlement, and that the government had to release a compensation of ₹4 lakhs within 24 h of being displaced by a katav”. The Thayillam collective on the other hand engages in assertions using art forms like performances of songs and dance that invokes historical and cultural memories of oppression and injustice. They also engage in other strategies of raising consciousness of oppression through public meetings, study classes, participation in movements, pamphlets and other publications.

### 4.5 Disaster recovery as an ongoing process of challenging everyday social relations, inequalities and injustices

In spite of experiencing recurrent disasters both communities have been able to make significant progress with regard to disaster recovery outcomes of gaining land, building homes, basic community amenities, accumulating assets, building disaster resilience and enhancing overall wellbeing. In 2007, the VMV movement was able to translate one of their goals into reality- 4 decimals of land for displaced families for rehabilitation. The Thayillam collective on the other hand, got wider local support and they could lease about 142 acres of paddy field for cultivation.

However, the two communities have been facing very different challenges in carrying forward the assertions embedded in their everyday lives and specific occasions of protest.

One of the key challenges facing VMV is with regard to the extremely poor socio-economic conditions and the travails emanating from the same. With the rampant migration of men most hamlets are left with only women, children and elderly. The educational levels of the Musahar communities that the VMV primarily attempts to mobilize are extremely low and this becomes a key challenge to building consciousness of oppression and rights among the affected communities. Thus, building a resistance movement and sustaining it sometimes seem to be a huge challenge as expressed by the members:

“Not all members are fully ready to sacrifice for the movement. Only few have evolved over the last 10 years, who are ready to even go to jail. This was not the case earlier. The process (i.e. being part of the movement) has instilled courage in them”.

Though both the initiatives strongly root themselves in non-violence and peaceful protest, they often are the target of atrocities from both the formal stakeholders and that of communities that surround them (especially in the case of VMV). Since the members of both initiatives negate any political affiliation with mainstream party politics, in an act of negating political society, they are often viewed with suspicion for affiliation with radical groups and they are under constant surveillance. The Thayillam members share their experience of being monitored as follows:

… Since we are associated with other tribal and environmental movements in the state, we were viewed with skepticism … … When we started rejecting government schemes and subsidies the skepticism increased …. and there was close surveillance on all our activities …. Though we have gained their confidence over the years, skepticism persists …

In the case of VMV despite following all protocols with regard to staging a protest, the members are often subjected to lathi-charge and precautionary lock up. Some have also been implicated with jail sentences on several occasions. They also undergo physical assaults and threats not just from the formal stakeholders but from fellow community members too. The landed groups feel threatened by the initiative as they often encroach large portions of Government land that has been allotted for resettlement of displaced communities. Thus, they try to thwart initiatives like that of VMV by offering large amounts to those who provide leadership or use physical threat and force to push back resistance of any kind.

Though VMV consciously detaches from its Dalit identity, they rarely escape the pain and injury inflicted by emotional and physical acts of aggression, violence, aversion and disgust in their everyday social relations that often spills over to the disaster recovery context. Their Dalit identity elicits resistance from among neighboring communities to prevent them from claiming land either encroached by the landed communities or anywhere near them. Often these encounters turn violent, in spite of the presence of police force and high-level officials at the scene. The failed attempts reinforce the pain and injury emerging from the everyday lived experiences of structural inequality and injustice, as expressed by one of the members after a violent clash with the neighboring communities:

… … We don't want to fight anymore … …. We have had enough … … …. .. I don't understand why we are left to live like this … … …. .. Everyone has so much of a disgust for us … … … …

Thus, these attempts to claim their rights often bring them closer to the realities of oppression and structural violence.

The Thayillam Collective does not face as much violent resistance as VMV from neighboring communities. The neighboring communities were oblivious of their presence or saw them to be of little significance in the neighborhood. However, with time, landowners are hesitant to lease land to the collective, as they perceive a threat of not being able to reclaim the land (for non-agricultural purposes) that has been recently leased out for farming purposes. It is a struggle for the Thayillam Collective to build lease relationship with the local land-owners and sustain it. They also face other challenges with regard to the lure of modernity among their own community members and that of the landed community around them. Though the current members of the collective recognize the politics of modernity and have rejected the same to be able to get back to their land and the way of life that is in sync with the environment and their belief system, they have their apprehensions with regard to the next generation. A large majority of the next generation have moved out in the pursuit of the so-called successes of the neoliberal world. Since agriculture in the wetland ecosystem is not an individualistic practice, the agricultural practices of agriculturalists around the Thayillam Collectives' fields are of grave significance to their long-term sustenance. The shift towards cash crops such as “oil palm” by elite investment agriculturalists from outside the region has been detrimental to the agricultural practices of the collective. These species are alien to the wetland ecosystem and they alter the pH balance to the detriment of the biodiversity that exist, and as of now there are no legal provisions to regulate the same. Above all the state backed encroachment of land by the elites have also led to the shrinking of rivers in the Kuttanad region of Kerala. This has hampered the fishing activities of inland fishers as their boats can no longer navigate the narrow river channels that have been encroached or blocked. The rivers have also become contaminated as they have become a dumping ground for both urban and agricultural waste. This has also intensified the destruction of biodiversity within the rivers. Portions of the Kayamkulam Lake were leveled or filled to construct a National Thermal Power Corporation plant and remaining areas had been dredged for the sake of creating boat ways for the National Inland Waterways, and the same has again hampered fishing activities. This has led to the destruction of the lake, a source of rich fish biodiversity, which in turn has destroyed the livelihood of both fishers and agriculturalist as they are synergistic in the given ecosystem.

## 5 Discussion and conclusion

The contradictions emerging from varied perspectives of the state or the formal actors and that of the community, about the conceptualization of disaster recovery and its operationalization, results in further marginalization of the communities at risk. Thus, mainstream recovery efforts never reduce vulnerabilities and injustice but reiterate these very structures of inequality. Disasters are being governed by both state and non-state formal agencies; hence every agency gets the liberty and freedom to define the affected community's life world. What agencies of recovery including the bureaucracy gain is the institutional freedom to define how recovery should be governed and implemented, however, such de facto jurisdiction does not engage with the recovery needs of the most marginalized communities and denies the right to mobilize and raise demands. The initial submission to agencies' institutional power is limited to a short period and with time the community start to recognize their rights to demand [44]. Such critical thinking is the reflection emerging from the multiple failed engagement with the political administration. The community assertions emerging from this precarious situation informs the alternate theoretical perspective on disaster recovery that aspire to address structural inequalities and injustices. Understanding pre-disaster structural inequalities and injustices, elucidates the power and structures of disaster recovery and alternate approaches to challenging them. An understanding of the power and structures of disaster recovery that emerges from the analysis of the grassroots assertions that challenge them comprises of the following three perspectives:

Firstly, disaster risk and recovery emerge from historical and everyday lived reality of marginalized communities, their social relations and resulting material conditions. The analysis of the two case studies demonstrate how the community's understanding of risk and recovery are embedded in their everyday life world and differ from that of the state and formal actors event centric perspective. It also highlights how the historically marginalized communities respond to these contradictions in the post disaster recovery context. The Vistapit Mukti Vahini and Thayillam are two classical movements which challenge the de facto power of agencies and bureaucracy to define the recovery process. Thus, recovery for structurally excluded communities is a political project of engaging with the state. Though these rights claim enable communities to externalize the pain and trauma of historical marginalization and deprivation, they seldom address the root causes that contribute to differential disaster risk and recovery outcomes among the most marginalized groups. Current institutional mechanisms too are oblivious to the structural constraints that these communities have historically experienced and its implication for exposure to disaster risk and recovery. Thus, these initiatives emerge from the belief that it is inevitable to fight the colonial pattern of land ownership through a collective assertion rather than seeking legal recourse. As the legal institutional mechanism has little to offer in terms of rights the two movements through their subversive practices address issue of structural inequalities and injustices that have implications for disaster risk and recovery. Thus, the two initiatives not only challenge the de-facto power of formal agencies, but also takes on the freedom to conceptualize risk within their life world and define recovery as grassroots level practices that address structural inequalities and injustices that contribute to differential exposure to risk and recovery outcomes.

Secondly, challenging everyday social relations, processes and injustices is central to the community's alternate conception and assertion for disaster recovery. Scholars of structural inequalities and injustice like Young [19] problematize the externalized identity and rights claim for their exclusive focus on the state (state policy, regulations, state institutions) from within a liberal framework that obscures the focus on civil society and the private sphere. The two case studies demonstrate how the movements do not restrict themselves to assertions against the state, but primarily challenge everyday social relations and processes within civil society and private spheres. Resistance, subversive and radical practices are embedded in their everyday livelihood, social and cultural practices, and are not just one-off events of rallying or protest. The movements also demonstrate the inevitability of ongoing assertions at the local everyday scale with the landowners and landlords, to retain the re-claimed land or agricultural practices, and further the recovery processes. The communities do not perceive recovery as an outcome to be achieved, but as an ongoing process of challenging everyday social relations, processes and injustices that they have historically encountered at all levels especially at the local and everyday scale. The communities do not conceptualize risk and recovery from within the disaster discourse alone, but as processes closely interlinked with the ecology, livelihood, culture and social relations that shape their everyday life world. Thus, the movement is not just a form of assertion for rights or identity claim, but a way of life and livelihood, based on the ideology of addressing lived experiences of structural inequalities and injustices not just in the post disaster recovery context, but in everyday life too. Often the injury of historical oppression is so deep that the movements often must start with the raising of consciousness among its members to even bring about the psychic resistance which seeks to shore up a notion of individual sovereignty against the shaping forces of history on our embodied lives [45]. Thus, the challenging of everyday social relations, processes and injustices often starts at the level of the individual, before moving onto other spheres.

Finally, community assertion and recovery rely on the mobilization of vulnerability, which could mean being exposed and agentic at the same time. The case studies negate the neo liberal subject position and calls into question the resilient or resisting body as discrete, singular and self-sufficient. It demonstrates the relational, dependent and dual nature of acts of resistance, which is very different from the idea of a political subject that establishes its agency by vanquishing its vulnerability, but as one that is exposed and agentic at the same time. The two cases demonstrate how the political subject becomes more vulnerable while trying to resist vulnerability to dispossession, poverty, insecurity and harm that constitutes a precarious position in the world, especially in a context where the subject is not endowed with that freedom as an inherent power or legitimacy, or when they live in a context of shrinking public space where open and supported movement is not possible. The cases also demonstrate the potential of resistance to address the material realities of vulnerability but highlights the struggles or barriers that it faces in exercising most basic rights (or the imperiled capacity to exercise its most basic right) when supportive environment falls apart or are emphatically unsupportive. The feminist critiques of neoliberalism [[3], [45], [47]] lament the shrinking of alternative imaginaries and are skeptical about acts of externalizing rights and identity claims as they lend limited possibility for political action, foregrounds deeper identification with one's marginality and dependence on the very oppressors [3]. However, the proponents of ontology of possibility [[29], [30], [31]] bring forth the possibility of subversively co-opting neo liberal rationalities to counter the vulnerabilities of being exposed. Cvetkovich [48] identifies counter cultural expression of trauma and injury as an alternative form of expression that prevent the internalization of the pain. Both these practices were evident in the two cases chosen for analysis.

To conclude, disaster creates maximum risk and causalities in the global south, and hence, state institutions are active in governing the disaster risks. In the global south, the vulnerable poor and marginalized groups are more exposed to disaster and in this critical context bureaucracy often takes control of the disaster recovery process too. Thus, like development projects, disaster recovery is also being governed by state institutions through bureaucratic regulations and control. Extending the same power on disaster recovery is being considered as ‘normal’, however such normal interventions often do not address the recovery needs of the most marginalized and triggers movements like the Vistapit Mukti Vahini and Thayillam. Structurally excluded communities perceive recovery as an instrument to overcome the institutional and political barriers on socio-economic mobility. Such inherent need for mobility influences the community involvement in recovery process. The two movements which are discussed in this paper are working towards building internal capability to overcome the structural issues which determine the recovery in post-disaster situation. It is a struggle to overcome the pre-disaster institutional influence on disaster recovery. So, these community assertions should not be read as just an alternative to the build back better approach to disaster recovery. These are movements demanding for distributive justice rather than the relative improvement conceived by the bureaucracy or the procedural pre-occupation among theories of justice. As theoretical perspectives on justice are primarily pre-occupied with procedural justice, especially in the context of disasters, in this paper we argue that theorizing on structural inequality and injustices in disaster recovery would benefit from the in depth focus on social relations and processes as they manifest in everyday lives of the most marginalized communities at risk.

## 6 Limitations

The collectives are not a homogeneous entity and might have inherent power dynamics and socio-cultural value systems that contribute to the persistence and exacerbation of structural inequalities. However, the study has not engaged with the internal dynamics of the collective and only looked at their interactions as a whole with the other. Thus, the primary data collection in limited to that of the key stakeholders and does not engage with every member of the collective. On similar lines the paper does not engage with the perspectives of the state or bureaucracy with regard the challenge of addressing structural inequalities and injustices.

## Funding

The research and preparation of manuscript was supported by the 10.13039/100000865Bill and Melinda Gates Foundation (OPP1130132) project “Disaster Resilience Leadership Academy (2017–19)”.

## Declaration of competing interest

The authors declare that they have no known competing financial interests or personal relationships that could have appeared to influence the work reported in this paper.
